# Income after cancer across gender and age among Canadian adolescents and young adults

**DOI:** 10.1093/jnci/djaf333

**Published:** 2025-11-19

**Authors:** Giancarlo Di Giuseppe, Arif Jetha, Petros Pechlivanoglou, Jason D Pole

**Affiliations:** Dalla Lana School of Public Health, University of Toronto, Toronto, ON, Canada; Child Health Evaluative Sciences, The Hospital for Sick Children Research Institute, Toronto, ON, Canada; Dalla Lana School of Public Health, University of Toronto, Toronto, ON, Canada; Institute for Work & Health, Toronto, ON, Canada; Child Health Evaluative Sciences, The Hospital for Sick Children Research Institute, Toronto, ON, Canada; Institute of Health Policy, Management and Evaluation, University of Toronto, Toronto, ON, Canada; Dalla Lana School of Public Health, University of Toronto, Toronto, ON, Canada; Centre for Health Services Research, University of Queensland, Brisbane, QLD, Australia

## Abstract

**Background:**

Cancer in adolescents and young adults emerges during critical transitional phases, resulting in lasting effects on financial well-being. It remains uncertain whether cancer in adolescents and young adults exhibits differences in financial impact on income based on gender and diagnosis age over time.

**Methods:**

We linked Canada’s national cancer registry to personal tax records to identify adolescents and young adults (aged 15-39 years) diagnosed between 1994 and 2013. In the year before diagnosis, survivors were variable-ratio matched to 10 cancer-free individuals on several sociodemographic characteristics. Participants were followed longitudinally up to 10-years postdiagnosis or until 2015. Relative and absolute income changes were estimated using doubly robust difference-in-differences. We categorized age into 3 groups: adolescents (aged 15-17 years), emerging young adults (aged 18-29 years), and young adults (aged 30-39 years), reflecting the different adolescent and young adult life stages. Analyses were stratified by gender and diagnosis age.

**Results:**

There were 60 240 women and 33 085 men survivors matched to 490 645 and 274 595 cancer-free participants, respectively. Overall, men and women had 6.9% (95% confidence interval [CI] = 5.1% to 8.6%) and 4.5% (95% CI = 3.1% to 5.8%) income reductions, respectively. Adolescent men had the largest reduction of 23.7% (95% CI = 1.9% to 40.6%), while a lack of statistical significance was observed in women of the same age. Income was reduced for varying magnitudes and durations across the different intersections of gender and diagnosis age, with men experiencing longer periods of income reductions.

**Conclusions:**

Cancer impacts income generation differently for adolescent and young adult men and women and at various diagnosis ages over time. Men, particularly younger men, are most vulnerable to income reductions.

## Introduction

Cancer in adolescents and young adults, typically defined as individuals aged 15-39 years, presents a unique clinical and public health challenge because of this critical life stage marked by the transition from childhood to adulthood.^1^ Adolescent teens transition from childhood into young adulthood as they pursue postsecondary education and enter the workforce. Meanwhile, emerging young adults (aged 20-29 years) complete their schooling and embark on their careers to achieve economic independence and a sense of identity.^2^ Young adults (aged 30-39 years) usually find themselves in the early stages of advancing their careers, starting families, and accumulating wealth. Because of this diverse developmental stage, adolescent and young adult survivors of cancer encounter distinct psychosocial and financial challenges compared with survivors from other age groups.^3-5^

However, the extent to which the intersection of gender and age at diagnosis affects financial impacts on survivorship is less understood compared with the general population that does not experience cancer during this developmental life stage. Cancer profoundly affects the financial well-being of adolescents and young adults by impacting short-term earning potential through absenteeism and treatment-related work disruptions, as well as long-term through reduced employment opportunities and diminished productivity.^6-10^ Qualitative studies among adolescent and young adult survivors demonstrate that financial effects vary across the diverse age range.^11^ Gender also plays a role in the financial implications of cancer because of differences in employment patterns, income levels, and career trajectories between men and women.^12^^,^^13^

Disparities in the life-course and societal roles may be intensified by cancer’s impact on employment and income among adolescents and young adults surviving cancer. For example, women may be thrust into caregiving roles or precarious employment situations that can lead to lower wages.^14^ Limited research has evaluated how diagnosis age, sex, and gender affect the financial well-being of adolescents and young adults.^15-17^ Yet, previous evidence has not assessed how financial challenges diverge at the intersection of gender and age. This has resulted in a knowledge gap regarding the financial implications of cancer on adolescents and young adults and the potential differences between men and women across various life stages.

Given these gaps, the primary aim of this study was to explore longitudinally the gender- and age-related differences in the impact of cancer on total income among adolescents and young adults within Canada’s universal health-care system. Specific objectives were to (1) estimate the impact of cancer on income for men and women at various life stages at the time of diagnosis and (2) assess short- and long-term implications for these groups. We hypothesized that gender would affect the financial impact differently and that age-related differences would also be observed.

## Methods

Ethics approval was obtained from the University of Toronto (Protocol #: 39870). This study adhered to the STROBE guidelines for reporting observational studies (Table S1).^18^ Informed consent was waived in accordance with Statistics Canada’s policies, as the study involved retrospective analysis of de-identified population-based administrative data.

### Study population

Details on cohort construction are described elsewhere.^19^ In brief, we identified adolescents and young adults aged 15-39 years diagnosed with malignant cancer from 1994 to 2013 using the Canadian Cancer Registry (CCR). The CCR is a national population-based registry capturing incident diagnoses among all residents of Canada.^20^ Records from the CCR were linked to Statistics Canada’s national income tax database from 1992 to 2015^21^ and to national immigration and mortality databases. We identified individuals never diagnosed with cancer using a 20% random sample from the same national tax database.

We individually matched each survivor to a variable number of individuals from the general population, up to a maximum of 10. In the year before diagnosis, we matched each survivor to participants on birth year, sex, marital status, children (yes, no), migration background (yes, no), census division geography, and income (within ±5%). Each cancer-free match was assigned a weight equal to the inverse total number of participants matched to their adolescent and young adult survivor.^22^^,^^23^

The index date for follow-up was the diagnosis date for survivors, and the same index date was assigned to their matches. We excluded survivors if geographic information at the time of diagnosis was unknown or if death or a secondary cancer diagnosis occurred within 2 years of their diagnosis. We followed participants longitudinally from the index date and censored them on the earliest occurrence of (1) death, (2) a second cancer, (3) lost to follow-up, or (4) achieved 10 years of follow-up or the end of data availability (December 31, 2015). Those censored on death or second cancer had income information up until, but not including, the year in which this censoring event occurred as we assumed these participants would be in their sickest health state.

### Measures

We captured gender (men and women) from self-reported information on the tax return and cancer registry. Diagnosis age was categorized into 15-17, 18-29, and 30-39 years to reflect adolescents, emerging young adults, and young adults, respectively. We chose this age categorization to reflect contemporary theoretical adolescent and young adult developmental life stages.^24^

The tax return provided information on family composition. Marital status (married, separated, or single or widowed) and number of children (0, 1, 2-3, or ≥4) were obtained. Geographic information was obtained from tax record postal codes, which were converted to neighborhood socioeconomic status income quintiles or a rural status.^25^

We obtained tumor characteristics to classify the major adolescent and young adult subtypes for descriptive reporting.^26^ We used Statistics Canada’s definition of total income to capture income information annually up to 5 years before index and until participant censoring, inflation adjusted to 2015 Canadian dollars.^27^

### Statistical analysis

All reported descriptive statistics were stratified by gender. We described continuous variables using means and standard deviations and categorical variables using frequencies and proportions. Differences in the distribution of characteristics between survivors and matched individuals were assessed using standardized mean differences.^28^ The variables used in the matching were described in the year before diagnosis to ensure comparability between cohorts before the cancer health shock.

We used a quasi-experimental study design using 2 groups (survivors and matched cancer-free individuals) and 2 periods (pre- and postdiagnosis) to quantify the potential effect of a cancer diagnosis on income. We implemented doubly robust difference-in-difference analysis to estimate the average income change prediagnosis to postdiagnosis for survivors compared with cancer-free matches.^29^^,^^30^ Difference-in-difference estimates were calculated for each follow-up year relative to the year preceding diagnosis to assess the changing impacts over time then combined for an aggregate summary measure.^30^ All regression analyses were stratified by gender to estimate differences in the financial impact of cancer, given the known disparities in employment patterns and wages between men and women. We then further stratified the difference-in-differences by age groupings to assess the financial impact across the developmental life stages.

Given the longitudinal nature of the data, missing income was imputed using multilevel predictive mean matching multiple imputation.^31^ We generated 5 imputed datasets and performed analyses on each set, pooling results. Analyses were performed using R (v4.1.2)^32^ at Statistics Canada. Bootstrapped 95% confidence intervals (CIs) were calculated for the difference-in-difference coefficients using 1000 replications.^30^ Two-tailed *P* values less than .05 were considered for statistical significance.

### Sensitivity analysis

We repeated the difference-in-difference analysis after removing the imputed data to assess our findings’ robustness and ensure that the imputation did not introduce bias.^33^

## Results

A total of 60 240 women and 33 085 men survivors were identified and matched with 490 645 and 274 595 cancer-free individuals, respectively. Population characteristics are presented in Table 1. An average of 8.1 years of follow-up was observed in both survivorship groups.

**Table 1.** Population characteristics of Canadian adolescent and young adult men and women diagnosed with cancer between 1994 and 2013^a^

Characteristic	Men			Women		
	AYA with cancer, %	Matched cancer-free AYA, %	Standardized mean difference	AYA with cancer, %	Matched cancer-free AYA, %	Standardized mean difference
Total sample size	33 085	274 595		60 240	490 645	
Age at index, y			0.009			0.011
Mean (SD)	30.9 (6.1)	30.9 (6.2)		32.6 (5.5)	32.6 (5.6)	
Adolescent (15-17)	1.3%	1.3%		0.6%	0.5%	
Emerging young adult (20-29)	37.0%	37.0%		25.7%	25.7%	
Young adult (30-39)	61.7%	61.7%		73.7%	73.7%	
Migration background	12.1%	12.1%	0.000	15.8%	15.8%	0.000
Canadian provincial region			0.001			0.000
Central	62.0%	62.0%		64.1%	64.1%	
Atlantic	7.0%	6.9%		6.9%	6.9%	
Prairies	18.2%	18.2%		17.2%	17.3%	
West Coast and Northern Territories	12.8%	12.8%		11.8%	11.8%	
Income quintile			0.027			0.027
Rural	16.5%	17.2%		15.6%	16.4%	
Quintile 1 (lowest)	15.9%	16.4%		16.4%	16.4%	
Quintile 2	16.5%	16.7%		16.3%	16.6%	
Quintile 3	16.6%	16.4%		16.8%	16.7%	
Quintile 4	17.5%	17.1%		17.9%	17.4%	
Quintile 5 (highest)	16.0%	15.5%		16.2%	15.8%	
(Missing)	1.0%	0.8%		0.8%	0.6%	
Marital status			0.000			0.000
Single or widowed	21.4%	21.4%		11.7%	11.7%	
Married or common-law	74.3%	74.3%		74.4%	74.4%	
Separated	4.2%	4.2%		13.9%	13.9%	
Children			0.012			0.011
0	39.6%	39.6%		27.4%	27.4%	
1	23.1%	22.7%		25.4%	25.1%	
2 or 3	34.2%	34.6%		42.9%	43.1%	
≥4	3.1%	3.2%		4.2%	4.4%	
Total income, CAD			0.002			0.002
Mean (SD)	$45 700 ($44 200)	$45 600 ($44 000)		$33 300 ($28 400)	$33 200 ($28 200)	
Censor reason			0.366			0.415
End of study	86.1%	92.9%		87.4%	95.7%	
Death	7.3%	0.6%		8.2%	0.4%	
Second cancer diagnosis	0.5%	0.0%		0.6%	0.0%	
Lost to follow-up	6.2%	6.5%		3.8%	3.9%	
Follow-up time, y			0.120			0.149
Mean (SD)	8.1 (3.0)	8.5 (2.8)		8.1 (2.9)	8.6 (2.7)	
0 to < 3	9.0%	6.9%		8.1%	5.9%	
3 to < 6	18.2%	15.6%		18.9%	15.4%	
6 to < 9	17.0%	16.3%		17.2%	16.3%	
≥9	55.9%	61.2%		55.7%	62.4%	
Cancer type						
Leukemias	5.7%			2.8%		
Lymphomas	19.1%			9.1%		
Central nervous system	6.1%			3.0%		
Sarcomas	5.2%			2.9%		
Blood and lymphatic vessel	0.8%			0.1%		
Nerve sheath	0.2%			0.1%		
Gonadal and related	25.8%			3.1%		
Melanoma	10.2%			9.6%		
Carcinomas	24.7%			66.5%		
Miscellaneous, specified	0.3%			0.3%		
Unspecified, except central nervous system	1.9%			2.6%		
Diagnosis era						
1994- 1999	29.4%			29.0%		
2000- 2004	25.0%			24.9%		
2005- 2009	26.2%			26.8%		
2010- 2013	19.4%			19.3%		

Abbreviation: AYA = adolescent and young adult.

a Descriptive statistics are weighted by the matching weight, except for the overall reported sample sizes. Family composition, total income, and income quintile are reported at the time of matching in the year before diagnosis.

Women were diagnosed at an older average (SD) age of 32.6 (5.6) years than the 30.9 (6.1) years observed in men. Among men, 1.3% were diagnosed as adolescents (15-17 years), 37.0% as emerging young adults (18-29 years), and 61.7% as young adults (30-39 years). For women, 0.6% were adolescents, 25.7% were emerging young adults, and 73.7% were young adults. The distribution of cancer subtypes differed between genders. Carcinomas were most diagnosed in women (66.5%), whereas gonadal tumors were most common among men (25.8%).

### Yearly average income throughout follow-up

**Figure 1.**
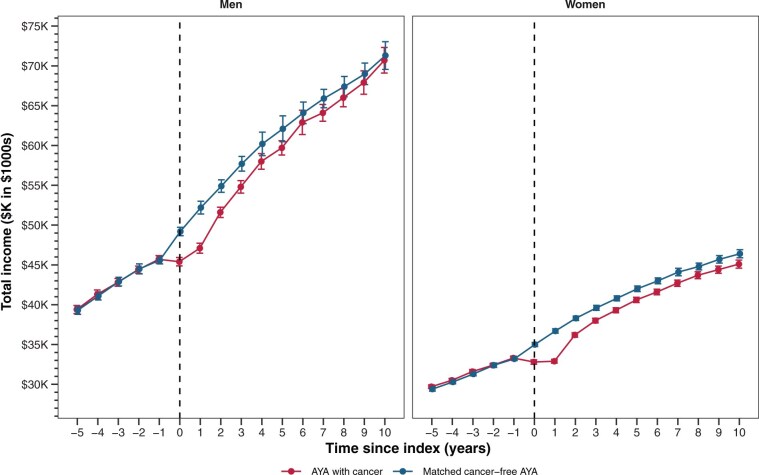
Income of adolescent and young adult (AYA) men and women survivors and matched cancer-free participants. Mean income with accompanying 95% confidence intervals, stratified for men and women, are reported longitudinally for each year of follow-up relative to the year of diagnosis (**dashed line**) for AYAs surviving cancer. Dollars are adjusted to 2015 Canadian dollars.

Figure 1 illustrates men had larger average income differences than women throughout most of the follow-up period. Variation in average income patterns was observed when stratified by gender and diagnosis age (Figure S1). Larger income differences occurred as the diagnosis age increased.

### Overall income reduction by gender

Table 2 presents the aggregated difference-in-difference results for men and women. Overall, cancer caused a 6.9% (95% CI = 5.1% to 8.6%) or $2438 (95% CI = $1673 to $3203) Canadian dollar income reduction in men. Women experienced a smaller income reduction of 4.5% (95% CI = 3.1% to 5.8%) or $1798 (95% CI = $1549 to $2047) Canadian dollars.

**Table 2.** Average impact of cancer on total income aggregated throughout study follow-up for Canadian adolescents and young adults stratified by gender and diagnosis age^a^

Diagnosis age	Absolute change ($, CAD)^b^			Percent change			*P*_pretrend_ ^c^
	Estimate	95% CI	*P*	Estimate	95% CI	*P*	
Men							
All ages combined	−$2438	(-$3203 to -$1673)	<.001	−6.9%	(-8.6% to -5.1%)	<.001	.353
Adolescent, 15-17 y	−$3356	(-$5346 to -$1366)	.001	−23.7%	(-40.6% to -1.9%)	.035	.828
Emerging young adult, 20-29 y	−$2562	(-$3229 to -$1894)	<.001	−7.6%	(-10.1% to -5.0%)	<.001	.625
Young adult, 30-39 y	−$2227	(-$3383 to -$1071)	<.001	−6.1%	(-8.3% to -3.8%)	<.001	.296
Women							
All ages combined	−$1798	(-$2047 to -$1549)	<.001	−4.5%	(-5.8% to -3.1%)	<.001	.102
Adolescent, 15-17 y	−$1609	(-$3078 to -$141)	.033	−16.7%	(-36.2% to 8.8%)	.180	.285
Emerging young adult, 20-29 y	−$1294	(-$1702 to -$886)	<.001	−4.7%	(-7.1% to -2.3%)	<.001	.495
Young adult, 30-39 y	−$1951	(-$2264 to -$1637)	<.001	−4.5%	(-6.0% to -2.9%)	<.001	.107

Abbreviation: CI = confidence interval.

a Estimates are provided for men and women separately and further stratified by age at diagnosis. Difference-in-difference estimates represent the average impact of cancer on income compared with matched cancer-free control participants.

b Dollar values are reported in 2015 Canadian dollars.

c Pretrend *P* >.05 indicates no difference in pretrends in income between adolescent and young adult survivors and matched cancer-free control participants.

### Overall income reduction by age and gender

Adolescent men experienced the largest income reduction of 23.7% (95% CI = 1.9% to 40.6%) or $3356 (95% CI = $1366 to $5346), while women of the same age had statistically insignificant relative reductions (Table 2). For the other age groups, income reductions ranged from 6.0% to 7.6% among men and were smaller for women, ranging from 4.5% to 4.9%. However, the 95% confidence intervals overlapped, suggesting no statistically significant differences by age.

### Longitudinal income changes by gender

**Figure 2.**
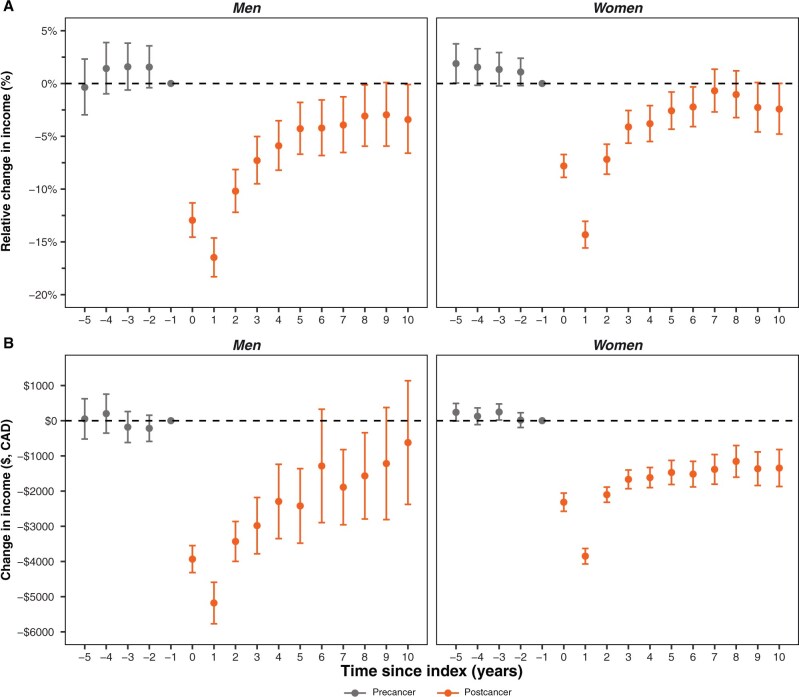
Temporal impact of cancer on total income for Canadian adolescents and young adults stratified by gender. **A**) Average percentage change in income. **B**) Absolute average dollar change inflation-adjusted to 2015 Canadian dollars. Difference-in-difference estimates provided are relative to the year before diagnosis and represent the average effect of cancer on income compared with matched cancer-free participants. **Dashed line** indicates the absence of an effect.

Men had larger magnitudes of relative (Figure 2, A) and absolute (Figure 2, B) income reductions than women over time. In the diagnosis year, men had 13.0% (95% CI = 11.3% to 14.6%) reduced income, whereas women had 7.8% (95% CI = 6.7% to 8.9%). At year 2 postdiagnosis, men had larger statistically significant reductions in income compared with women, equaling 10.2% (95% CI = 8.1% to 12.2%) and 7.2% (95% CI = 5.8% to 8.6%), respectively. By the 5th year postdiagnosis, income reductions were similar across gender.

### Longitudinal income changes by age and gender

**Figure 3.**
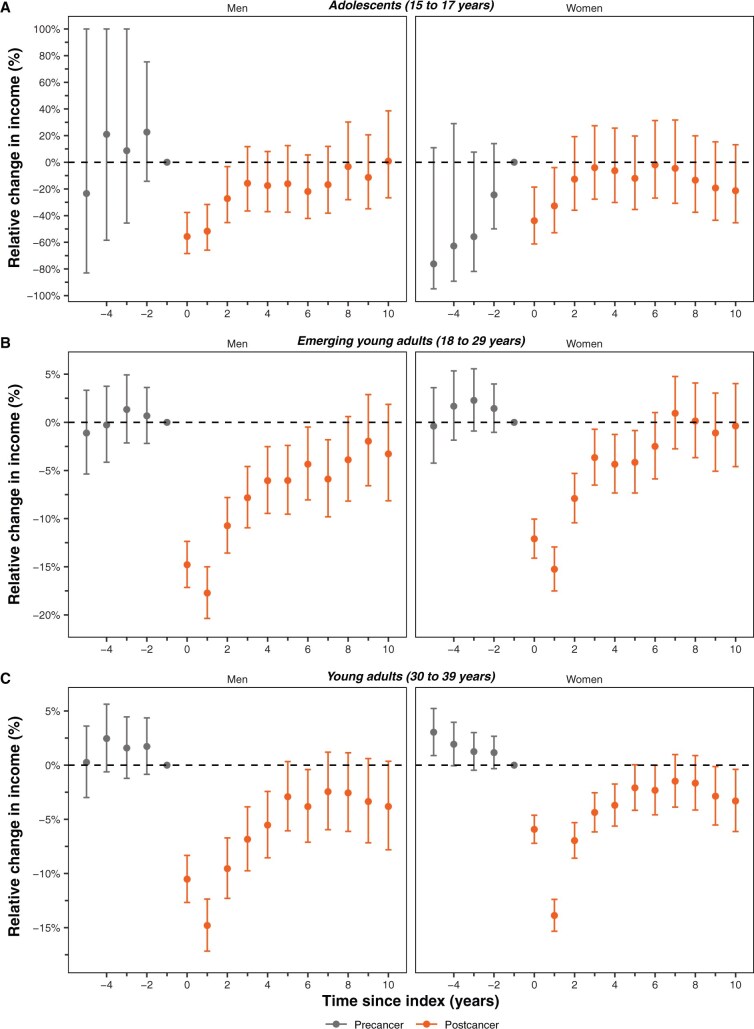
Yearly percentage changes in total income for Canadian adolescents and young adults with cancer stratified by men and women. **A**) Adolescents (age 15-17 years) at diagnosis, **(B**) emerging young adults (age 18-29 years) at diagnosis, and **(C**) young adults (age 30-39 years) at diagnosis. Estimates provided are relative to the year before diagnosis and represent the average effect of cancer compared with matched cancer-free participants. **Dashed line** indicates the absence of an effect.

The magnitude and pattern of income reduction varied across age and gender over time for percentage (Figure 3) and absolute dollar (Figure S2) income changes, with full details also provided in Table S2. Within the first few years after diagnosis, the largest income reductions occurred in the younger age groups for men and women. In the diagnosis year for adolescents, men and women had 55.7% (95% CI = 37.7% to 45.6%) and 31.7% (95% CI = 22.1% to 40.2%) reductions in income, respectively. In this same period, men and women had 10.5% and 5.9% reductions in the young adult group, respectively. Income reductions attenuated over time for all groups. Adolescents experienced the largest reductions but also recovered the quickest, while emerging young adults had the longest periods of sustained income reductions.

Men had statistically significant percentage income losses for longer periods of time than women. For example, among emerging young adults, changes in income for women were no longer statistically significant by the 6th year postdiagnosis, whereas this occurred by the 8th year for men (Figure 3, B). This pattern occurred for all groups except young adults, where men recovered their absolute dollar changes by the 6th year postdiagnosis, whereas women never did (Figure S2C).

### Sensitivity analyses

Results were consistent in the complete case analysis for the aggregated (Table S3) and yearly (Table S4) difference-in-differences analyses.

## Discussion

Our study illustrates that cancer adversely affects total income to varying degrees between men and women, as well as across different stages of life at diagnosis. Men experience greater income reductions, which are sustained for longer than those of women. Critical periods of financial vulnerability exist for adolescents and young adults with cancer, with younger men at the highest risk of experiencing financial strain. These findings highlight the significance of gender and the timing of a cancer diagnosis in the life course when considering the financial implications of this disease.

Despite Canada’s universal health-care system, which does not require direct payment for medically necessary treatment, we demonstrate the indirect financial consequences that survivors face following their cancer diagnosis by measuring income losses. In countries without universal coverage, the financial hardships of cancer are often exacerbated by the direct costs of medical care, leading to even greater economic distress among survivors.^34-36^ Adolescents and young adults in those countries face challenges with health literacy, insurance coverage, and high out-of-pocket costs, often leading to a considerable financial burden and delaying or forgoing care.^37-40^ In many ways, our findings may reflect a more conservative scenario for survivors than what is observed in other health-care systems where the financial impact may be more severe. Nonetheless, the persistence of income losses among survivors within a publicly funded system underscores that universal health care alone does not protect against the broader economic consequences of cancer.

Our results have important implications for supporting targeted financial interventions, tailored to meet the needs of adolescents and young adults, throughout various stages of the cancer care continuum.^41^^,^^42^ Discussions surrounding financial navigation, costs of care, and financial counseling may help alleviate immediate and long-term financial toxicities.^43-45^ This is crucial for adolescents or emerging young adults who are at a greater risk of facing financial distress, as they may lack the financial literacy, resources, or savings necessary to buffer the economic impacts of their illness. Adolescent and young adult survivors frequently express a need for financial assistance and support, often reporting unmet financial needs and hardships.^46-48^ However, our findings suggest that the financial distress faced by survivors remains unaddressed, and future research and policy development are warranted to ameliorate challenges faced by adolescents and young adults.

We illustrate gender differences in financial impacts, with men experiencing greater income reductions associated with cancer. This finding is consistent with a 2022 longitudinal study examining labor market earnings in adult male cancer survivors.^49^ A possible explanation is that men may begin their careers early and have higher earning potential, which may result in a greater financial impact after cancer. Furthermore, adolescents and young adults in their late twenties and thirties are typically starting families and may face additional related financial pressures. In Canada, national maternity and parental leave benefits are provided through a federal insurance program that offers partial income replacement for eligible workers who have accumulated sufficient insurable hours based on previous employment. Consequently, younger survivors or those without established work histories may receive insufficient support. Some employers provide top-up benefits, but coverage is inconsistent and varies across industries and workplaces. These factors may partly explain the financial strain observed among adolescent and young adult survivors, particularly those in the family-building life stage.

Cancer contributes to substantial disruptions to employment and work ability.^50-52^ If these cancer-related disruptions occur early in the career trajectory, they may have lasting implications on future earnings. This is consistent with the life course perspective, which suggests that the timing of health shocks can have long-lasting impacts on future outcomes.^53^ Men and women cancer survivors exhibit different patterns of psychosocial stress and work ability, which can impact the ability to earn income.^54-56^ Future research should explore the mechanisms underlying the implications of cancer during the critical career developmental adolescent and young adult years and how these impacts may differ between genders.

Income reductions were largest in the first few years following diagnosis for all intersections of gender and age. This initial income reduction, followed by a recovery, aligns with the U-shaped recovery pattern observed in other studies of cancer survivors within universal health-care systems.^49^ The younger age groups had the largest declines, with men exhibiting a slower recovery, yet adolescents appeared to recover financially more quickly than emerging young adults. This may suggest that experiencing a diagnosis as an emerging young adult, especially for men, when careers are becoming established but not fully formed may have a considerable impact on future earnings, whereas in adolescents, where career trajectories are less defined and more precarious, this may allow for a quicker recovery. It is not until the oldest age group where similarities between genders are observed, coinciding with a time of job stability and career advancement. This employment security and advanced career stage in older adolescents and young adults may act as a buffer against the financial distress younger survivors experience. Future research should delve deeper into identifying the specific roles that career stage, employment precarity, and potential support systems have in shaping these age-related financial trajectories after cancer.

This study is strengthened by the linkage of Canada’s national cancer registry to personal income tax returns to provide a comprehensive and population-based sample of adolescents and young adults diagnosed with cancer. Using this data linkage, this is the largest study to date to examine the intersection of gender and age at diagnosis on the financial outcomes of adolescents and young adults surviving cancer. We longitudinally followed survivors over a 10-year period, allowing for the dynamic capture of short- and long-term financial impacts of cancer, particularly for those who are at different stages of life at diagnosis.

Several study limitations must be considered. Results should be interpreted with caution as some statistically significant findings may be because of chance given the number of stratified analyses. Data on disease severity, treatments received, or comorbidities were unavailable, and these factors are associated with worse financial outcomes among cancer survivors.^57-59^ Restricting inclusion to participants surviving at least 2 years after diagnosis may have introduced survivorship bias, and as a result, our findings may underestimate the financial impact because those who died shortly after diagnosis may experience the greatest financial distress. Although we constructed a matched cohort to be as similar as possible to the survivors, time-varying confounders such as changes in marital status, employment status, mental health, or other life events were not accounted for in the analysis and may influence the results. Additionally, the absence of occupational classification data limited our ability to assess the types of jobs participants held and how these may have influenced income. Lastly, we were limited to tax information for the individual and could not account for household income, identification as a dependent, or other sources of unreported financial support. In such instances, parental and/or spousal support is unmeasured, and with our results unable to account for this, we may be overestimating the financial distress experienced by certain survivor groups.

In conclusion, cancer impacts the total income of adolescent and young adult men and women with a varying degree and across different stages of life at diagnosis. Income reductions are largest in the years following cancer diagnosis, with a U-shaped recovery pattern observed during the 10-year follow-up period. Men experience greater reductions in total income resulting from cancer, and recovery is generally slower. Future studies should explore the mechanisms underlying the implications of cancer during the critical career development years of adolescents and young adults at different life stages. Targeted financial interventions are needed to support survivors experiencing financial distress after their cancer diagnosis.

## Supplementary Material

djaf333_Supplementary_Data

## Data Availability

Data used in this project were provided by Statistics Canada and accessed through one or more of the RDCs (Research Data Centre) in the Canadian Research Data Centre Network (CRDCN). Because of the confidential nature of these microdata, they cannot be shared. Researchers in Canada working at one of CRDCN’s member institutions can access the data at no additional cost to the researcher. Other researchers will have to pay cost-recovery to access the data. Access to the data is subject to a background check and research approval process. The protocols for data access, including fees for researchers at non-CRDCN institutions, can be found on the CRDCN website (https://crdcn.ca/publications-data/access-crdcn-data/).
