# Supplementary material for: Income after cancer across gender and age among Canadian adolescents and young adults
Source: J Natl Cancer Inst. 2025 Nov 19;118(3):492–501. doi: 10.1093/jnci/djaf333 (PMC13017703; doi:10.1093/jnci/djaf333)
Supplement: djaf333_Supplementary_Data [file djaf333_supplementary_data.pdf]

Supplementary Material

for

Income After Cancer Across Gender and Age Among Canadian Adolescents and Young Adults

Giancarlo Di Giuseppe, PhD, MPH[1,2,*]; Arif Jetha, PhD[1,3]; Petros Pechlivanoglou, PhD[2,4]; Jason D. Pole, PhD[1,5]

[1]Dalla Lana School of Public Health, University of Toronto, Toronto, ON, Canada
[2]Child Health Evaluative Sciences, The Hospital for Sick Children Research Institute, Toronto, ON, Canada
[3]Institute for Work & Health, Toronto, ON, Canada
[4]Institute of Health Policy, Management and Evaluation, University of Toronto, Toronto, ON, Canada
[5]Centre for Health Services Research, University of Queensland, Brisbane, QLD, Australia

List of Supplementary Tables

S1 STROBE Statement–Checklist of items that should be included in reports of cohort studies . . . 2
S2 Yearly relative and absolute changes in total income for Canadian adolescents and young adults with cancer stratified by gender and diagnosis age . . . 4
S3 Complete case aggregated difference-in-difference estimates of the impact of cancer on total income among working-aged adolescents and young adults in Canada stratified by gender and diagnosis age . . . 6
S4 Complete case analysis of the gender- and age-stratified difference-in-difference estimates for the yearly impact of cancer on total income among working-aged adolescents and young adults in Canada . . . 7

List of Supplementary Figures

S1 Gender and diagnosis age stratified income for AYA survivors and matched cancer-free participants . . . 10
S2 Yearly dollar changes in total income for Canadian adolescents and young adults with cancer stratified by men and women . . . 11

Table S1: STROBE Statement–Checklist of items that should be included in reports of cohort studies

	Item No.	Recommendation	Page No.
**Title and abstract**	1	(a) Indicate the study's design with a commonly used term in the title or the abstract	Title & Abstract
		(b) Provide in the abstract an informative and balanced summary of what was done and what was found	Abstract
**Introduction**			
Background / rational	2	Explain the scientific background and rationale for the investigation being reported	Introduction, para 1-3
Objectives	3	State specific objectives, including any prespecified hypotheses	Introduction, para 4
**Methods**			
Study design	4	Present key elements of study design early in the paper	*Study population*, para 1-3
Setting	5	Describe the setting, locations, and relevant dates, including periods of recruitment, exposure, follow-up, and data collection	*Study population*, para 1
Participants	6	(a) Give the eligibility criteria, and the sources and methods of selection of participants. Describe methods of follow-up	*Study population*, para 2-3
		(b) For matched studies, give matching criteria and number of exposed and unexposed	*Study population*, para 2
Variables	7	Clearly define all outcomes, exposures, predictors, potential confounders, and effect modifiers. Give diagnostic criteria, if applicable	*Measures*, para 1-3
Data sources / measurement	8	For each variable of interest, give sources of data and details of methods of assessment (measurement). Describe comparability of assessment methods if there is more than one group	*Study population*, para 1; *Measures*, para 1-3
Bias	9	Describe any efforts to address potential sources of bias	*Study population*, para 1-2; *Sensitivity analysis*
Study size	10	Explain how the study size was arrived at	Provided in Methods, para 1 reference to previous study
Quantitative variables	11	Explain how quantitative variables were handled in the analyses. If applicable, describe which groupings were chosen and why	*Statistical analysis*, para 1
Statistical methods	12	(a) Describe all statistical methods, including those used to control for confounding	*Statistical analysis*, para 2-3
		(b) Describe any methods used to examine subgroups and interactions	*Statistical analysis*, para 2
		(c) Explain how missing data were addressed	*Statistical analysis*, para 3
		(d) If applicable, explain how loss to follow-up was addressed	*Study population*, para 3
		(e) Describe any sensitivity analyses	*Sensitivity analyses*
**Results**			
Participants	13	(a) Report numbers of individuals at each stage of study—eg numbers potentially eligible, examined for eligibility, confirmed eligible, included in the study, completing follow-up, and analysed	Results, para 1
		(b) Give reasons for non-participation at each stage	Provided in Methods, para 1 reference to previous study
		(c) Consider use of a flow diagram	
Descriptive data	14	(a) Give characteristics of study participants (eg demographic, clinical, social) and information on exposures and potential confounders	Table 1
		(b) Indicate the number of participants with missing data for each variable of interest	
		(c) Summarize baseline characteristics	
Outcome data	15	Report numbers of outcome events or summary measures over time	Figure 1 & 2
Main results	16	(a) Give unadjusted estimates and, if applicable, confounder-adjusted estimates and their precision (eg, 95% confidence interval). Make clear which confounders were adjusted for and why they were included	Table 2; Results, para 4-6
		(b) Report category boundaries when continuous variables were categorized	
		(c) If relevant, consider translating estimates of relative risk into absolute risk for a meaningful time period	
Other analyses	17	Report other analyses done—eg analyses of subgroups and interactions, and sensitivity analyses	Figure 3; Results, para 7-9
**Discussion**			
Key results	18	Summarize key results with reference to study objectives	Discussion, para 1

Continued on the next page

	Item No.	Recommendation	Page No.
Limitations	19	Discuss limitations of the study, taking into account sources of potential bias or imprecision. Discuss both direction and magnitude of any potential bias	*Strengths and limitations*, para 2
Interpretation	20	Give a cautious overall interpretation of results considering objectives, limitations, multiplicity of analyses, results from similar studies, and other relevant evidence	Discussion, para 3-6
Generalizability	21	Discuss the generalizability (external validity) of the study results	Discussion, para 2 & *Strengths and limitations*, para 2
**Other information**			
Funding	22	Give the source of funding and the role of the funders for the present study and, if applicable, for the original study on which the present article is based	*Funding*

From: von Elm E, Altman DG, Egger M, Pocock SJ, Gøtzsche PC, Vandenbroucke JP. The Strengthening the Reporting of Observational Studies in Epidemiology (STROBE) statement: guidelines for reporting observational studies. *Lancet.* 2007;370(9596):1453–1457. doi:10.1016/S0140-6736(07)61602-X

Table S2: Yearly relative and absolute changes in total income for Canadian adolescents and young adults with cancer stratified by gender and diagnosis age

	Men				Women			
	Absolute ($)		*Relative (%)*		*Absolute ($)*		*Relative (%)*	
**Time**	**Estimate (95% CI)**	**P-value**	**Estimate (95% CI)**	**P-value**	**Estimate (95% CI)**	**P-value**	**Estimate (95% CI)**	**P-value**
All ages combined								
-5	$52 (-$520 to $625)	0.858	-0.4% (-3.0% to 2.3%)	0.792	$241 (-$9 to $490)	0.058	1.9% (0.0% to 3.8%)	0.044
-4	$202 (-$352 to $755)	0.475	1.4% (-1.0% to 3.9%)	0.250	$126 (-$111 to $362)	0.297	1.6% (-0.2% to 3.3%)	0.077
-3	-$180 (-$620 to $260)	0.421	1.6% (-0.6% to 3.8%)	0.161	$249 ($20 to $477)	0.033	1.3% (-0.2% to 2.9%)	0.096
-2	-$217 (-$589 to $155)	0.253	1.6% (-0.4% to 3.6%)	0.120	$17 (-$193 to $227)	0.874	1.1% (-0.2% to 2.4%)	0.098
-1	Reference		Reference		Reference		Reference	
0	-$3,932 (-$4,316 to -$3,549)	<0.001	-13.0% (-14.6% to -11.3%)	<0.001	-$2,315 (-$2,574 to -$2,057)	<0.001	-7.8% (-8.9% to -6.7%)	<0.001
1	-$5,180 (-$5,770 to -$4,589)	<0.001	-16.5% (-18.3% to -14.6%)	<0.001	-$3,850 (-$4,071 to -$3,629)	<0.001	-14.3% (-15.6% to -13.0%)	<0.001
2	-$3,430 (-$3,997 to -$2,864)	<0.001	-10.2% (-12.2% to -8.1%)	<0.001	-$2,101 (-$2,317 to -$1,886)	<0.001	-7.2% (-8.6% to -5.8%)	<0.001
3	-$2,980 (-$3,780 to -$2,181)	<0.001	-7.3% (-9.5% to -5.0%)	<0.001	-$1,666 (-$1,931 to -$1,400)	<0.001	-4.1% (-5.6% to -2.6%)	<0.001
4	-$2,294 (-$3,350 to -$1,239)	<0.001	-5.9% (-8.2% to -3.5%)	<0.001	-$1,616 (-$1,902 to -$1,330)	<0.001	-3.8% (-5.5% to -2.1%)	<0.001
5	-$2,420 (-$3,479 to -$1,362)	<0.001	-4.3% (-6.7% to -1.8%)	<0.001	-$1,470 (-$1,812 to -$1,128)	<0.001	-2.6% (-4.3% to -0.8%)	0.004
6	-$1,285 (-$2,896 to $326)	0.118	-4.2% (-6.8% to -1.6%)	0.002	-$1,516 (-$1,879 to -$1,154)	<0.001	-2.2% (-4.1% to -0.3%)	0.022
7	-$1,889 (-$2,958 to -$820)	<0.001	-3.9% (-6.5% to -1.3%)	0.004	-$1,382 (-$1,803 to -$960)	<0.001	-0.7% (-2.7% to 1.4%)	0.505
8	-$1,567 (-$2,792 to -$342)	0.012	-3.1% (-5.9% to -0.1%)	0.041	-$1,154 (-$1,604 to -$705)	<0.001	-1.0% (-3.2% to 1.2%)	0.359
9	-$1,217 (-$2,809 to $375)	0.134	-3.0% (-5.9% to 0.1%)	0.057	-$1,364 (-$1,842 to -$886)	<0.001	-2.3% (-4.6% to 0.1%)	0.059
10	-$621 (-$2,378 to $1,135)	0.488	-3.4% (-6.6% to -0.1%)	0.044	-$1,344 (-$1,870 to -$817)	<0.001	-2.4% (-4.8% to 0.0%)	0.050
15 to 17 years at diagnosis								
-5	-$843 (-$6,750 to $5,064)	0.780	-23.4% (-83.0% to 244.9%)	0.729	-$5,293 (-$11,859 to $1,274)	0.113	-76.2% (-94.9% to 10.9%)	0.068
-4	-$9 (-$5,381 to $5,363)	0.997	21.0% (-58.5% to 252.6%)	0.727	-$2,992 (-$8,730 to $2,746)	0.296	-62.8% (-89.3% to 29.0%)	0.119
-3	$353 (-$2,557 to $3,263)	0.811	8.7% (-45.6% to 117.2%)	0.813	-$2,246 (-$3,750 to -$741)	0.004	-55.8% (-81.9% to 7.6%)	0.072
-2	-$380 (-$1,851 to $1,091)	0.612	22.6% (-14.3% to 75.3%)	0.264	-$1,811 (-$2,710 to -$913)	<0.001	-24.4% (-49.9% to 14.0%)	0.182
-1	Reference		Reference		Reference		Reference	
0	-$1,167 (-$2,824 to $490)	0.165	-55.7% (-68.5% to -37.7%)	<0.001	-$1,766 (-$3,295 to -$237)	0.026	-43.8% (-61.2% to -18.6%)	0.002
1	-$2,099 (-$3,617 to -$580)	0.007	-51.7% (-65.9% to -31.6%)	<0.001	-$994 (-$2,549 to $561)	0.210	-32.7% (-52.9% to -4.0%)	0.029
2	-$3,082 (-$4,622 to -$1,542)	<0.001	-27.2% (-45.2% to -3.2%)	0.029	-$1,323 (-$3,075 to $429)	0.136	-12.6% (-36.0% to 19.3%)	0.395
3	-$1,752 (-$3,897 to $393)	0.109	-15.8% (-36.5% to 11.8%)	0.235	-$290 (-$2,681 to $2,101)	0.808	-4.0% (-27.7% to 27.4%)	0.777
4	-$3,655 (-$6,301 to -$1,009)	0.008	-17.5% (-37.0% to 8.1%)	0.163	-$668 (-$2,393 to $1,057)	0.445	-6.3% (-30.1% to 25.6%)	0.663
5	-$4,024 (-$6,815 to -$1,233)	0.005	-16.1% (-37.4% to 12.5%)	0.240	-$805 (-$5,556 to $3,946)	0.734	-12.0% (-35.4% to 19.7%)	0.415
6	-$5,970 (-$9,211 to -$2,729)	<0.001	-21.9% (-42.1% to 5.5%)	0.107	-$481 (-$2,915 to $1,953)	0.698	-2.0% (-26.8% to 31.3%)	0.894
7	-$5,248 (-$8,890 to -$1,607)	0.005	-16.8% (-38.1% to 11.9%)	0.224	-$420 (-$3,666 to $2,826)	0.799	-4.5% (-30.7% to 31.7%)	0.779
8	-$4,538 (-$8,207 to -$868)	0.015	-3.2% (-28.0% to 30.2%)	0.829	-$2,554 (-$5,971 to $863)	0.142	-13.4% (-37.5% to 19.9%)	0.385
9	-$664 (-$11,271 to $9,943)	0.902	-11.4% (-34.8% to 20.6%)	0.443	-$4,114 (-$8,408 to $180)	0.060	-19.3% (-43.5% to 15.3%)	0.239
10	-$4,718 (-$9,603 to $168)	0.058	0.8% (-26.6% to 38.6%)	0.959	-$4,289 (-$8,416 to -$162)	0.042	-21.4% (-45.4% to 13.2%)	0.196
18 to 29 years at diagnosis								

Continued on next page

	Men				Women			
	Absolute ($)		*Relative (%)*		*Absolute ($)*		*Relative (%)*	
**Time**	**Estimate (95% CI)**	**P-value**	**Estimate (95% CI)**	**P-value**	**Estimate (95% CI)**	**P-value**	**Estimate (95% CI)**	**P-value**
-5	$384 (-$581 to $1,348)	0.435	-1.1% (-5.4% to 3.3%)	0.618	$50 (-$353 to $453)	0.806	-0.4% (-4.2% to 3.6%)	0.844
-4	$178 (-$716 to $1,071)	0.691	-0.3% (-4.1% to 3.7%)	0.892	$6 (-$346 to $359)	0.972	1.7% (-1.9% to 5.3%)	0.355
-3	-$88 (-$555 to $380)	0.713	1.3% (-2.1% to 4.9%)	0.456	$219 (-$128 to $567)	0.214	2.3% (-0.9% to 5.6%)	0.162
-2	-$67 (-$515 to $382)	0.766	0.7% (-2.2% to 3.6%)	0.646	$56 (-$249 to $360)	0.713	1.4% (-1.0% to 4.0%)	0.258
-1	Reference		Reference		Reference		Reference	
0	-$3,904 (-$4,313 to -$3,494)	<0.001	-14.8% (-17.1% to -12.4%)	<0.001	-$2,296 (-$2,567 to -$2,026)	<0.001	-12.1% (-14.1% to -10.0%)	<0.001
1	-$4,518 (-$5,299 to -$3,737)	<0.001	-17.7% (-20.4% to -15.0%)	<0.001	-$2,932 (-$3,303 to -$2,560)	<0.001	-15.3% (-17.5% to -12.9%)	<0.001
2	-$3,193 (-$3,832 to -$2,555)	<0.001	-10.7% (-13.6% to -7.8%)	<0.001	-$1,640 (-$2,017 to -$1,262)	<0.001	-7.9% (-10.4% to -5.3%)	<0.001
3	-$2,901 (-$3,692 to -$2,111)	<0.001	-7.8% (-11.0% to -4.6%)	<0.001	-$1,253 (-$1,690 to -$816)	<0.001	-3.7% (-6.5% to -0.7%)	0.015
4	-$2,276 (-$3,079 to -$1,473)	<0.001	-6.1% (-9.5% to -2.5%)	<0.001	-$1,283 (-$1,764 to -$803)	<0.001	-4.3% (-7.3% to -1.3%)	0.006
5	-$2,120 (-$2,950 to -$1,289)	<0.001	-6.0% (-9.5% to -2.4%)	0.001	-$1,220 (-$1,815 to -$624)	<0.001	-4.2% (-7.3% to -0.9%)	0.014
6	-$1,858 (-$2,884 to -$831)	<0.001	-4.3% (-8.1% to -0.5%)	0.028	-$1,032 (-$1,625 to -$440)	<0.001	-2.5% (-5.9% to 1.0%)	0.162
7	-$2,026 (-$3,156 to -$897)	<0.001	-5.9% (-9.8% to -1.8%)	0.005	-$788 (-$1,477 to -$99)	0.025	0.9% (-2.7% to 4.8%)	0.622
8	-$1,586 (-$2,813 to -$359)	0.011	-3.9% (-8.2% to 0.6%)	0.089	-$723 (-$1,464 to $19)	0.056	0.1% (-3.7% to 4.1%)	0.943
9	-$1,850 (-$3,209 to -$491)	0.008	-2.0% (-6.6% to 2.9%)	0.420	-$523 (-$1,329 to $282)	0.203	-1.1% (-5.1% to 3.0%)	0.598
10	-$1,944 (-$3,616 to -$272)	0.023	-3.3% (-8.2% to 1.9%)	0.207	-$546 (-$1,468 to $375)	0.244	-0.4% (-4.6% to 4.0%)	0.864
30 to 39 years at diagnosis								
-5	-$36 (-$770 to $699)	0.924	0.3% (-3.0% to 3.6%)	0.878	$359 ($49 to $670)	0.023	3.0% (0.9% to 5.2%)	0.005
-4	$300 (-$433 to $1,033)	0.423	2.5% (-0.6% to 5.6%)	0.118	$221 (-$69 to $510)	0.135	1.9% (0.0% to 4.0%)	0.056
-3	-$208 (-$820 to $403)	0.504	1.6% (-1.2% to 4.5%)	0.269	$284 ($9 to $558)	0.043	1.3% (-0.5% to 3.0%)	0.155
-2	-$307 (-$811 to $198)	0.234	1.7% (-0.8% to 4.4%)	0.189	$31 (-$244 to $306)	0.828	1.2% (-0.3% to 2.7%)	0.125
-1	Reference		Reference		Reference		Reference	
0	-$4,008 (-$4,562 to -$3,454)	<0.001	-10.5% (-12.7% to -8.3%)	<0.001	-$2,328 (-$2,666 to -$1,990)	<0.001	-5.9% (-7.2% to -4.6%)	<0.001
1	-$5,631 (-$6,507 to -$4,755)	<0.001	-14.8% (-17.2% to -12.4%)	<0.001	-$4,192 (-$4,454 to -$3,930)	<0.001	-13.9% (-15.3% to -12.4%)	<0.001
2	-$3,537 (-$4,407 to -$2,668)	<0.001	-9.5% (-12.3% to -6.7%)	<0.001	-$2,268 (-$2,534 to -$2,002)	<0.001	-7.0% (-8.6% to -5.3%)	<0.001
3	-$2,977 (-$4,234 to -$1,721)	<0.001	-6.8% (-9.8% to -3.8%)	<0.001	-$1,807 (-$2,136 to -$1,478)	<0.001	-4.4% (-6.2% to -2.5%)	<0.001
4	-$2,122 (-$3,786 to -$459)	0.012	-5.5% (-8.5% to -2.4%)	<0.001	-$1,715 (-$2,086 to -$1,345)	<0.001	-3.7% (-5.6% to -1.7%)	<0.001
5	-$2,382 (-$4,013 to -$751)	0.004	-2.9% (-6.1% to 0.3%)	0.079	-$1,530 (-$1,940 to -$1,119)	<0.001	-2.1% (-4.2% to 0.0%)	0.054
6	-$637 (-$3,133 to $1,858)	0.617	-3.8% (-7.1% to -0.4%)	0.029	-$1,655 (-$2,095 to -$1,214)	<0.001	-2.3% (-4.6% to 0.0%)	0.050
7	-$1,536 (-$3,213 to $140)	0.072	-2.4% (-6.0% to 1.2%)	0.186	-$1,561 (-$2,069 to -$1,053)	<0.001	-1.5% (-3.9% to 1.0%)	0.237
8	-$1,317 (-$3,109 to $476)	0.150	-2.6% (-6.1% to 1.1%)	0.173	-$1,260 (-$1,811 to -$708)	<0.001	-1.7% (-4.1% to 0.9%)	0.200
9	-$666 (-$3,075 to $1,743)	0.588	-3.4% (-7.2% to 0.6%)	0.097	-$1,590 (-$2,177 to -$1,002)	<0.001	-2.9% (-5.5% to -0.1%)	0.041
10	$320 (-$2,249 to $2,890)	0.807	-3.8% (-7.8% to 0.4%)	0.073	-$1,552 (-$2,192 to -$913)	<0.001	-3.3% (-6.1% to -0.4%)	0.027

Effects represent the impact of cancer on income compared with matched cancer-free participants. Dollar values are reported in 2015 Canadian dollars. *Abbreviations:* 95% confidence interval (95%CI), 95% confidence interval.

Table S3: Complete case aggregated difference-in-difference estimates of the impact of cancer on total income among working-aged adolescents and young adults in Canada stratified by gender and diagnosis age

	**Absolute Change ($, CAD)**			**Relative Change (%)**			
**Diagnosis age**	**Estimate**	**95% CI**	**P-value**	**Estimate**	**95% CI**	**P-value**	**Pretrend P-value**
Men							
All ages combined	-$2,763	(-$3,555 to -$1,972)	<0.001	-7.5%	(-9.2% to -5.7%)	<0.001	0.385
15 - 17	-$3,622	(-$5,556 to -$1,689)	<0.001	-22.1%	(-41.0% to -4.7%)	0.041	0.890
18 - 29	-$2,816	(-$3,524 to -$2,109)	<0.001	-7.9%	(-10.7% to -5.8%)	<0.001	0.660
30 - 39	-$2,535	(-$3,706 to -$1,364)	<0.001	-6.3%	(-8.8% to -4.2%)	<0.001	0.342
Women							
All ages combined	-$1,819	(-$2,066 to -$1,572)	<0.001	-4.4%	(-5.8% to -3.0%)	<0.001	0.051
15 - 17	-$1,491	(-$2,874 to -$107)	0.035	-15.1%	(-36.7% to 10.4%)	0.247	0.731
18 - 29	-$1,300	(-$1,705 to -$895)	<0.001	-4.6%	(-7.0% to -2.3%)	<0.001	0.361
30 - 39	-$1,968	(-$2,270 to -$1,666)	<0.001	-4.3%	(-5.9% to -2.9%)	<0.001	0.139

Estimates are provided for men and women separately, and further stratified by age at diagnosis. Difference-in-difference estimates represent the average impact of cancer on income compared with matched cancer-free participants. Dollar values are reported in 2015 Canadian dollars. Pretrend P-values with values > 0.05 indicate no difference in pre-trends in income between adolescent and young adult survivors and matched cancer-free participants. *Abbreviations:* 95%CI, 95% confidence interval.

Table S4: Complete case analysis of the gender- and age-stratified difference-in-difference estimates for the yearly impact of cancer on total income among working-aged adolescents and young adults in Canada

	Men		Women	
	Absolute ($)	***Relative (%)***	***Absolute ($)***	***Relative (%)***
**Time**	**Estimate (95% CI)**	**Estimate (95% CI)**	**Estimate (95% CI)**	**Estimate (95% CI)**
All ages combined				
-5	$95 (-$672 to $863)	0.0% (-3.7% to 4.0%)	$310 (-$22 to $643)	2.2% (-0.6% to 5.0%)
-4	$156 (-$606 to $919)	1.7% (-1.9% to 5.4%)	$185 (-$137 to $507)	1.8% (-0.7% to 4.4%)
-3	-$175 (-$728 to $379)	1.9% (-1.3% to 5.2%)	$247 (-$44 to $537)	1.5% (-0.6% to 3.6%)
-2	-$216 (-$720 to $287)	1.9% (-0.7% to 4.6%)	$39 (-$241 to $318)	1.3% (-0.5% to 3.1%)
-1	Reference	Reference	Reference	Reference
0	-$4,146 (-$4,667 to -$3,625)	-13.4% (-15.6% to -11.2%)	-$2,356 (-$2,709 to -$2,004)	-7.8% (-9.4% to -6.3%)
1	-$5,515 (-$6,307 to -$4,723)	-17.2% (-19.7% to -14.7%)	-$3,873 (-$4,147 to -$3,599)	-14.4% (-16.2% to -12.5%)
2	-$3,813 (-$4,650 to -$2,975)	-11.1% (-13.9% to -8.2%)	-$2,122 (-$2,424 to -$1,819)	-7.2% (-9.3% to -5.0%)
3	-$3,332 (-$4,470 to -$2,195)	-8.0% (-11.2% to -4.6%)	-$1,672 (-$2,050 to -$1,293)	-4.0% (-6.2% to -1.8%)
4	-$2,608 (-$4,110 to -$1,107)	-6.4% (-9.6% to -3.0%)	-$1,623 (-$2,036 to -$1,211)	-3.7% (-6.1% to -1.3%)
5	-$2,715 (-$4,322 to -$1,108)	-4.8% (-8.1% to -1.4%)	-$1,480 (-$1,926 to -$1,035)	-2.5% (-5.0% to 0.1%)
6	-$1,626 (-$4,082 to $829)	-4.8% (-8.3% to -1.1%)	-$1,537 (-$2,042 to -$1,033)	-2.1% (-4.8% to 0.6%)
7	-$2,208 (-$3,925 to -$492)	-4.6% (-8.3% to -0.8%)	-$1,401 (-$2,015 to -$786)	-0.6% (-3.3% to 2.3%)
8	-$1,952 (-$3,719 to -$185)	-3.6% (-7.7% to 0.6%)	-$1,172 (-$1,851 to -$494)	-1.0% (-4.0% to 2.2%)
9	-$1,536 (-$3,849 to $777)	-3.4% (-7.3% to 0.7%)	-$1,395 (-$2,093 to -$698)	-2.1% (-5.5% to 1.4%)
10	-$941 (-$3,497 to $1,615)	-3.6% (-8.0% to 1.0%)	-$1,376 (-$2,120 to -$632)	-2.4% (-6.0% to 1.4%)
15 to 17 years at diagnosis				
-5	-$629 (-$5,218 to $3,960)	-24.9% (-92.3% to 628.1%)	-$4,009 (-$6,979 to -$1,040)	-71.7% (-96.7% to 144.1%)
-4	-$295 (-$4,371 to $3,780)	20.4% (-73.4% to 445.6%)	-$2,687 (-$5,074 to -$300)	-61.6% (-93.1% to 112.7%)
-3	$324 (-$2,625 to $3,273)	7.8% (-58.5% to 180.1%)	-$1,534 (-$3,241 to $174)	-54.9% (-87.3% to 59.9%)
-2	-$168 (-$1,326 to $990)	25.3% (-23.4% to 105.0%)	-$1,288 (-$2,181 to -$394)	-22.0% (-56.3% to 39.2%)
-1	Reference	Reference	Reference	Reference
0	-$1,945 (-$2,716 to -$1,173)	-58.9% (-74.7% to -33.1%)	-$1,199 (-$2,053 to -$345)	-42.0% (-64.1% to -6.2%)
1	-$2,341 (-$3,682 to -$1,001)	-53.8% (-71.1% to -26.1%)	-$1,331 (-$2,760 to $98)	-34.9% (-59.6% to 4.8%)
2	-$2,891 (-$4,861 to -$920)	-27.2% (-50.5% to 6.9%)	-$816 (-$2,398 to $766)	-11.0% (-41.7% to 35.9%)
3	-$2,435 (-$4,952 to $81)	-17.5% (-43.2% to 19.8%)	$176 (-$2,040 to $2,392)	-2.6% (-34.4% to 44.8%)
4	-$4,113 (-$7,036 to -$1,189)	-19.7% (-44.7% to 16.7%)	-$239 (-$2,397 to $1,918)	-4.6% (-37.6% to 45.7%)
5	-$3,994 (-$7,326 to -$661)	-16.4% (-42.7% to 22.0%)	-$674 (-$3,367 to $2,018)	-12.9% (-43.8% to 34.9%)
6	-$5,899 (-$10,114 to -$1,684)	-21.1% (-47.9% to 19.5%)	-$494 (-$3,646 to $2,657)	-1.9% (-34.6% to 47.2%)

Continued on next page

Time	Men		Women	
	Absolute ($)	***Relative (%)***	***Absolute ($)***	***Relative (%)***
	**Estimate (95% CI)**	**Estimate (95% CI)**	**Estimate (95% CI)**	**Estimate (95% CI)**
7	-$5,555 (-$10,551 to -$558)	-18.0% (-45.1% to 22.4%)	-$436 (-$4,447 to $3,576)	-4.9% (-38.8% to 47.5%)
8	-$4,440 (-$9,431 to $551)	-3.5% (-35.9% to 45.2%)	-$3,007 (-$7,268 to $1,255)	-13.5% (-45.2% to 36.4%)
9	-$771 (-$15,392 to $13,849)	-11.1% (-40.4% to 32.6%)	-$4,115 (-$9,271 to $1,041)	-19.7% (-51.0% to 31.4%)
10	-$5,462 (-$12,167 to $1,242)	-0.7% (-35.8% to 53.7%)	-$4,261 (-$10,185 to $1,663)	-21.0% (-52.8% to 32.0%)
18 to 29 years at diagnosis				
-5	$553 (-$809 to $1,915)	-0.6% (-6.3% to 5.4%)	$151 (-$358 to $660)	0.1% (-5.5% to 6.0%)
-4	$268 (-$637 to $1,173)	0.5% (-5.1% to 6.5%)	$157 (-$317 to $630)	2.4% (-2.4% to 7.4%)
-3	$21 (-$510 to $551)	1.9% (-3.1% to 7.2%)	$269 (-$133 to $671)	2.7% (-1.6% to 7.3%)
-2	$34 (-$414 to $483)	1.2% (-2.9% to 5.4%)	$77 (-$217 to $371)	1.7% (-2.0% to 5.5%)
-1	Reference	Reference	Reference	Reference
0	-$4,013 (-$4,434 to -$3,591)	-15.2% (-18.3% to -12.0%)	-$2,311 (-$2,622 to -$2,001)	-12.2% (-14.9% to -9.4%)
1	-$4,742 (-$5,825 to -$3,658)	-18.5% (-21.9% to -15.0%)	-$2,905 (-$3,342 to -$2,468)	-15.3% (-18.5% to -12.1%)
2	-$3,438 (-$4,322 to -$2,555)	-11.5% (-15.7% to -7.1%)	-$1,625 (-$2,153 to -$1,096)	-7.9% (-11.6% to -4.2%)
3	-$3,094 (-$4,190 to -$1,997)	-8.5% (-12.7% to -4.0%)	-$1,199 (-$1,832 to -$566)	-3.4% (-7.8% to 1.2%)
4	-$2,486 (-$3,665 to -$1,307)	-6.6% (-11.2% to -1.8%)	-$1,249 (-$1,940 to -$558)	-4.2% (-8.4% to 0.1%)
5	-$2,253 (-$3,309 to -$1,198)	-6.6% (-11.3% to -1.7%)	-$1,218 (-$1,976 to -$460)	-4.1% (-8.8% to 0.7%)
6	-$2,134 (-$3,474 to -$795)	-4.9% (-10.2% to 0.7%)	-$1,038 (-$1,923 to -$154)	-2.4% (-7.5% to 3.0%)
7	-$2,308 (-$3,893 to -$724)	-6.9% (-12.4% to -1.1%)	-$817 (-$1,739 to $105)	1.0% (-4.6% to 7.0%)
8	-$1,991 (-$3,775 to -$207)	-4.8% (-10.7% to 1.6%)	-$723 (-$1,799 to $353)	0.2% (-5.6% to 6.3%)
9	-$2,224 (-$4,362 to -$85)	-2.8% (-8.8% to 3.7%)	-$574 (-$1,701 to $552)	-1.1% (-7.3% to 5.4%)
10	-$2,295 (-$4,555 to -$36)	-3.4% (-10.0% to 3.8%)	-$638 (-$1,914 to $638)	-0.5% (-7.2% to 6.7%)
30 to 39 years at diagnosis				
-5	-$100 (-$1,051 to $850)	0.5% (-3.8% to 4.9%)	$432 ($21 to $844)	3.4% (0.4% to 6.4%)
-4	$199 (-$827 to $1,225)	2.5% (-1.6% to 6.7%)	$265 (-$82 to $611)	2.2% (-0.7% to 5.1%)
-3	-$239 (-$1,073 to $596)	1.9% (-2.1% to 6.0%)	$274 (-$117 to $665)	1.4% (-1.3% to 4.1%)
-2	-$338 (-$1,060 to $383)	2.1% (-1.4% to 5.8%)	$54 (-$315 to $423)	1.4% (-0.9% to 3.7%)
-1	Reference	Reference	Reference	Reference
0	-$4,278 (-$5,086 to -$3,471)	-11.0% (-13.9% to -8.0%)	-$2,370 (-$2,831 to -$1,910)	-5.9% (-7.6% to -4.2%)
1	-$6,003 (-$7,229 to -$4,778)	-15.5% (-18.7% to -12.1%)	-$4,210 (-$4,558 to -$3,862)	-13.9% (-16.2% to -11.6%)
2	-$3,955 (-$5,208 to -$2,703)	-10.5% (-14.1% to -6.7%)	-$2,285 (-$2,658 to -$1,912)	-7.0% (-9.4% to -4.5%)
3	-$3,372 (-$5,208 to -$1,536)	-7.5% (-11.9% to -2.9%)	-$1,820 (-$2,249 to -$1,390)	-4.3% (-7.1% to -1.4%)

Continued on next page

Time	Men: Absolute ($) Estimate (95% CI)	Men: Relative (%) Estimate (95% CI)	Women: Absolute ($) Estimate (95% CI)	Women: Relative (%) Estimate (95% CI)
4	-$2,406 (-$4,844 to $32)	-5.8% (-10.1% to -1.4%)	-$1,724 (-$2,204 to -$1,244)	-3.6% (-6.4% to -0.8%)
5	-$2,692 (-$5,187 to -$197)	-3.4% (-8.2% to 1.7%)	-$1,538 (-$2,065 to -$1,011)	-2.0% (-5.1% to 1.2%)
6	-$897 (-$4,832 to $3,039)	-4.2% (-8.9% to 0.8%)	-$1,675 (-$2,305 to -$1,045)	-2.2% (-5.6% to 1.3%)
7	-$1,791 (-$4,305 to $724)	-2.9% (-7.8% to 2.3%)	-$1,573 (-$2,281 to -$864)	-1.3% (-4.9% to 2.4%)
8	-$1,633 (-$4,255 to $988)	-2.8% (-7.8% to 2.4%)	-$1,277 (-$2,020 to -$534)	-1.6% (-5.6% to 2.6%)
9	-$908 (-$4,529 to $2,713)	-3.5% (-8.8% to 2.0%)	-$1,611 (-$2,441 to -$780)	-2.6% (-6.7% to 1.6%)
10	$53 (-$3,763 to $3,868)	-4.0% (-9.7% to 2.0%)	-$1,562 (-$2,525 to -$600)	-3.2% (-7.2% to 0.9%)

Effects represent the impact of cancer on income compared with matched cancer-free participants. Dollar values are reported in 2015 Canadian dollars. *Abbreviations:* 95%CI, 95% confidence interval.

Figure S1: Gender and diagnosis age stratified income for AYA survivors and matched cancer-free participants

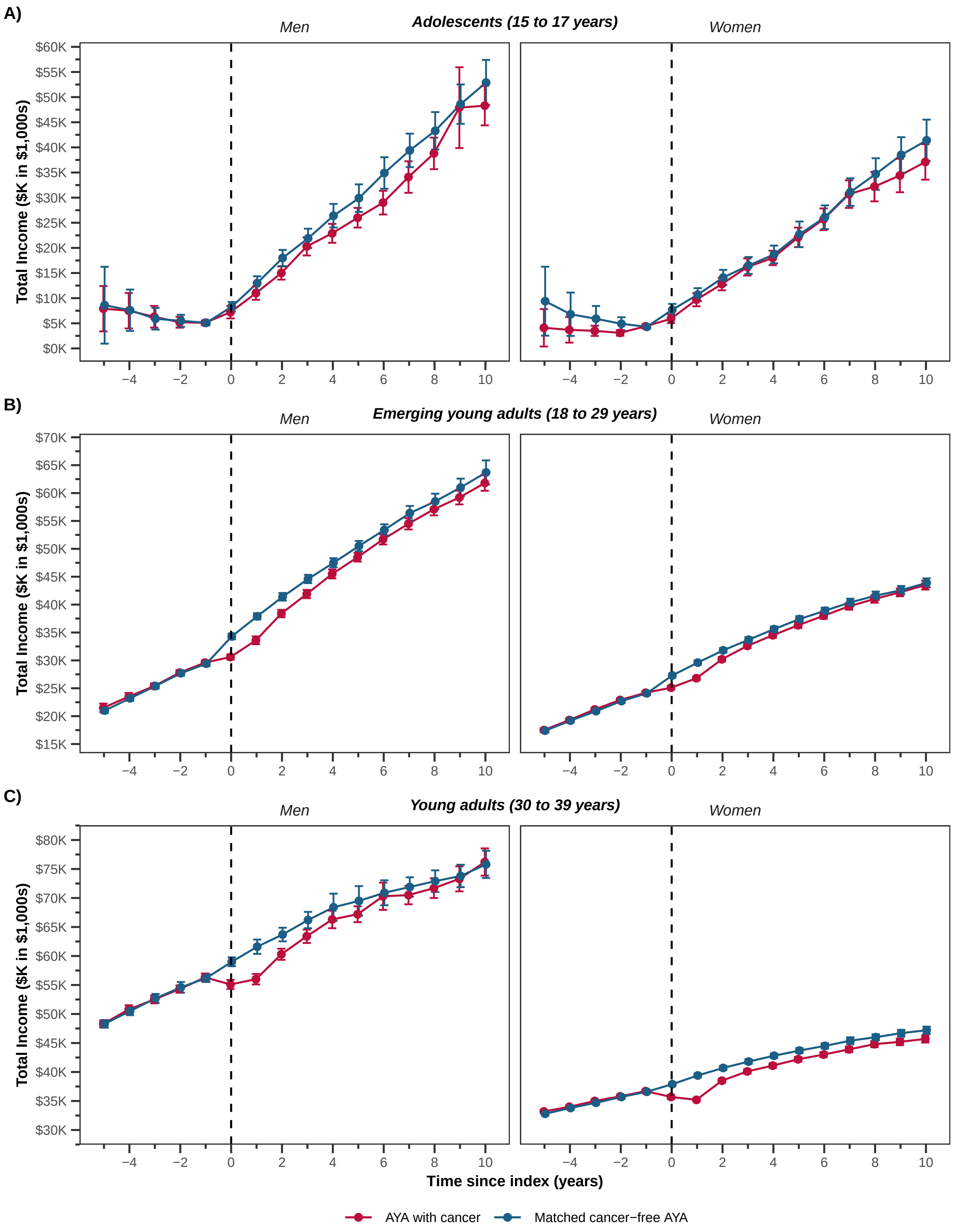

Dashed line represents the year of diagnosis for adolescents and young adults with cancer. Dollars are adjusted to 2015 Canadian dollars.

Figure S2: Yearly dollar changes in total income for Canadian adolescents and young adults with cancer stratified by men and women

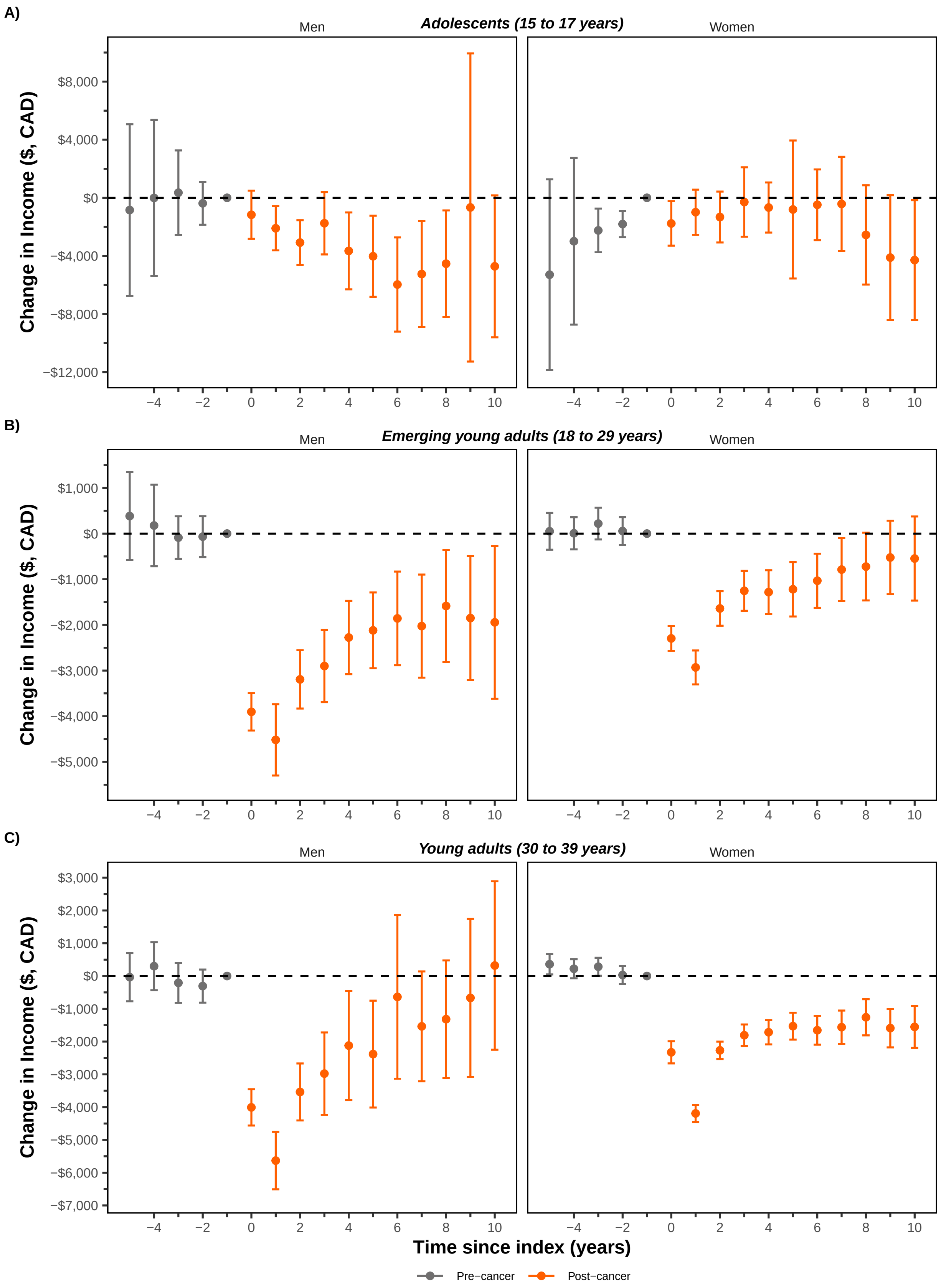

A) Adolescents (15–17 years) at diagnosis, B) Emerging young adults (18–29 years) at diagnosis, and C) Young adults (30–39 years) at diagnosis. Difference-in-difference estimates provided are relative to the year before diagnosis and represent the average effect of cancer compared with matched cancer-free participants. Dashed line indicates the absence of an effect. Dollars are inflation-adjusted to 2015 Canadian dollars.
